# Phytochemical basis, multitarget pharmacology, and translational perspectives of silymarin: emphasis on its antifibrotic effects

**DOI:** 10.3389/fphar.2026.2001949

**Published:** 2026-10-23

**Authors:** Zhe Jin, Jing Zhu, Binghong Lu, Yabo Li, Yan Chen

**Affiliations:** Department of Otolaryngology, Jingjiang People’s Hospital Affiliated to Yangzhou University, Jingjiang, Jiangsu, China

**Keywords:** fibrosis, flavonolignans, natural product pharmacology, Silybum marianum, silymarin, TGF-β/Smad signaling

## Abstract

This review critically evaluates the antifibrotic potential of silymarin by integrating its phytochemical composition, formulation-dependent exposure, molecular pharmacology, cross-organ preclinical evidence, and clinical translation, while distinguishing multicomponent silymarin extracts from purified silybin/silibinin and delivery-enhanced preparations. A structured, non-systematic evidence-mapping search of PubMed/MEDLINE, Web of Science, and Scopus covered English-language literature through December 2025. A targeted update of PubMed/MEDLINE and publisher records was performed through 9 August 2026, focusing on fibrosis, pharmacokinetics, formulation, drug delivery, ferroptosis, succinate metabolism, ANXA6, and related mechanisms. Preclinical studies indicate that silymarin-related interventions can modulate TGF-β/Smad signaling, oxidative and inflammatory pathways, fibroblast activation, myofibroblast differentiation, and extracellular-matrix turnover. Recent work has added hepatocyte succinate handling, FTH1-related ferroptosis, and ANXA6-associated stress signaling. Antifibrotic activity has been reported in liver, kidney, lung, peritoneal, and skin models, whereas human evidence remains limited and heterogeneous. Product composition, oral exposure, formulation, and endpoint selection may contribute to discordant clinical findings. Silymarin has biologically plausible multitarget antifibrotic activity, but current evidence does not establish it as an antifibrotic therapy. Future studies should use chemically defined preparations, exposure-informed dosing, validated target engagement, and fibrosis-centered outcomes.

## Graphical Abstract

**Figure:**
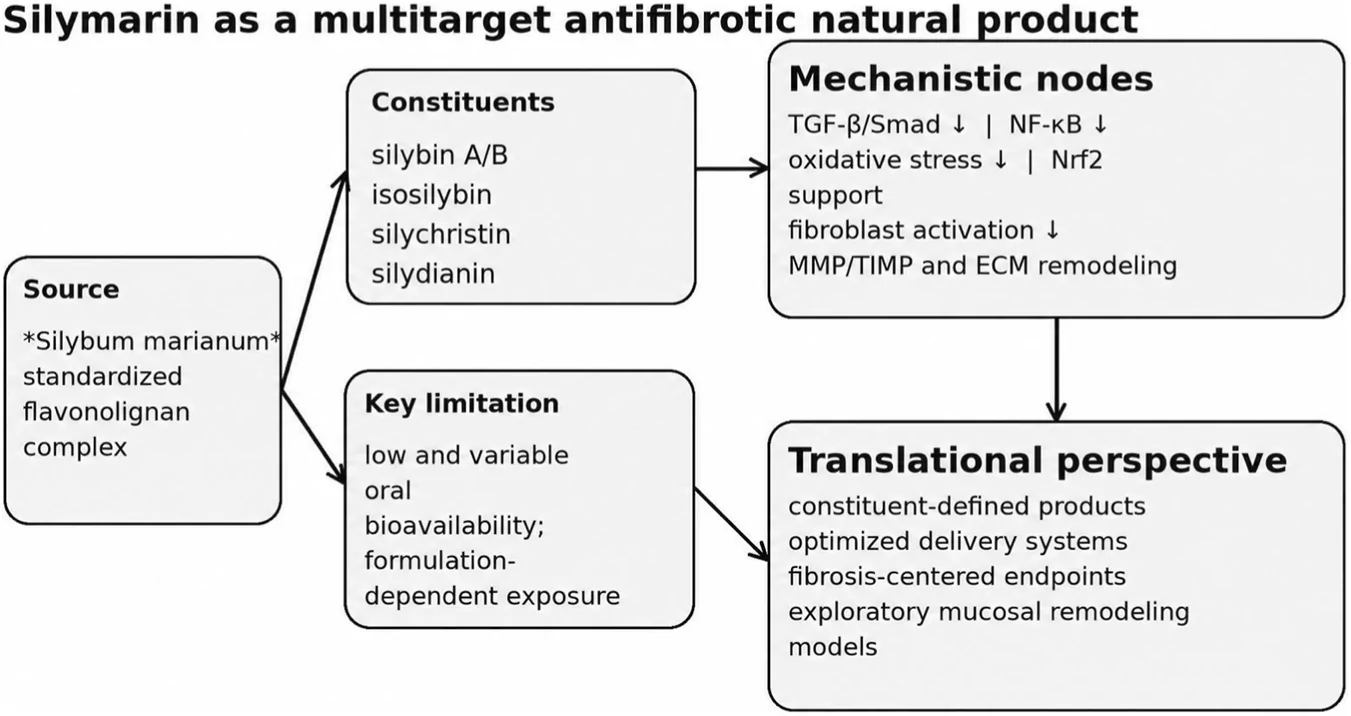

## 1 Introduction

Fibrosis develops when persistent tissue injury drives maladaptive repair and progressive extracellular-matrix accumulation, ultimately compromising organ structure and function. Importantly, fibrotic change is not invariably permanent: in chronic liver disease, regression can occur after the causal injury is removed or effectively treated (Iredale et al., 1998; Rockey and Friedman, 2021). In MASLD, the extent of fibrosis is also closely linked to subsequent liver-related outcomes (Lam et al., 2024; Younossi et al., 2026).

Therapeutic progress in MASH further shows that disease activity and fibrotic burden can be pharmacologically modified. This is clearly illustrated in the Phase 3 data for semaglutide and resmetirom, where complete fibrosis improvement is seen in only a subset of treated patients (Sanyal et al., 2025; Harrison et al., 2024). This residual disease burden provides room for other therapies that can target interconnected inflammatory and fibrogenic processes.

Silymarin is a flavonolignan-rich natural product derived from Silybum marianum (Křen and Valentová, 2022; Chen et al., 2024). HPLC profiling has revealed that a number of characteristic flavonolignan ratios are very consistent from one commercial extract to another (Chen et al., 2024); however, the overall composition of a preparation may vary due to processing, plant chemotype and source (Chambers et al., 2017). Experimental studies indicate a role in the inhibition of hepatic stellate-cell activation and associated myofibroblastic response (Fuchs et al., 1997; Ezhilarasan et al., 2016) and purified silibinin inhibits the proliferation and migration of LX-2 hepatic stellate cells (Ezhilarasan et al., 2016). Translation is more complicated for several reasons, including low and variable systemic exposure with conventional oral preparations (Zhu et al., 2013) and the absence of consistent clinical effects in randomized trials of chronic hepatitis C (Fried et al., 2012).

Several recent reviews overlap with parts of this topic. Wu et al. (Wu et al., 2024) discussed silybin-based approaches and targeted delivery in liver disease; Sayyad et al. (Sayyad et al., 2025) reviewed mechanisms, pharmacokinetics, delivery technologies, and preclinical applications; Liu et al. (Liu et al., 2025) focused on silybin in hepatic fibrosis; and Li et al. (Li X. et al., 2025) examined biopharmaceutical limitations and newer delivery strategies. Our review takes a different approach by organizing the evidence around five questions that are directly relevant to translation: what intervention was tested; whether the achieved or expected exposure was pharmacologically plausible; how strong the mechanistic evidence was; at what stage of fibrosis treatment was initiated; and whether the outcome directly measured fibrosis or relied on a surrogate endpoint. This framework helps explain why extensive preclinical activity has not yet translated into consistent clinical antifibrotic efficacy.

The key question is not whether silymarin is already an established antifibrotic therapy, but whether the available evidence on composition, standardization, exposure, mechanism, preclinical efficacy, and human studies justifies further development in fibrosis. We therefore use these domains to identify priorities for constituent-defined and formulation-controlled research. Throughout the review, “antifibrotic” is used as an umbrella term only when the underlying evidence category is clear; prevention or attenuation of fibrogenesis, treatment of established fibrosis, and regression of pre-existing scar are not considered equivalent outcomes.

## 2 Evidence acquisition

We used a structured, non-systematic evidence-mapping approach to identify experimental and clinical literature on silymarin, silibinin, and fibrosis-related biology. The core search included PubMed/MEDLINE, Web of Science, and Scopus databases, searching for English articles until December 2025, using search terms including milk thistle, Silybum marianum, silymarin, silibinin, fibrosis, fibrogenesis, extracellular matrix, TGF-β, Smad, oxidative stress, inflammation, NASH, MASH, MASLD, diabetic nephropathy, and nasal polyps. This was followed by a targeted update in PubMed/MEDLINE and the publisher records through 9 August 2026 with new keywords (pulmonary fibrosis, renal fibrosis, ferroptosis, endoplasmic reticulum stress, succinate, ANXA6, drug delivery, nanocarrier, liposome, and bioavailability). The electronic search results were supplemented by reference lists of the identified relevant publications that were searched for eligible studies.

Eligible evidence comprised *in vitro* studies, animal studies, and human studies that assessed silymarin or one of its defined components in fibroblast or hepatic stellate-cell activation, collagen accumulation, ECM remodeling, TGF-β/Smad signaling, oxidative or inflammatory signaling, ferroptosis, metabolic signaling, direct target engagement, or clinically relevant fibrosis-associated outcomes. Orientation and context were provided through reviews and editorials while the main anchors for mechanistic and clinical inferences were the original studies.

This was a critical narrative review rather than a formal systematic review; therefore, no PRISMA flow diagram was used. The synthesis emphasizes clear study identification, careful interpretation across evidence types, and source-to-claim correspondence, consistent with guidance for narrative reviews (Baethge et al., 2019). Duplicate records across databases were reconciled using title, authorship, publication year, and DOI when available. Because no formal quality score was applied, evidence was weighted qualitatively. Human randomized studies and studies with fibrosis-specific structural endpoints were given greater translational weight than studies relying mainly on biochemical surrogates. Mechanistic claims were considered stronger when supported by target-binding, loss- or gain-of-function, rescue, or pathway-perturbation experiments. Conflicting findings were retained and examined, particularly when differences in preparation, fibrosis stage, dose, formulation, or endpoint could plausibly explain the discrepancy. This approach was intended to make the basis for our interpretation transparent while preserving the narrative-review design.

## 3 Evidence synthesis

### 3.1 Phytochemical composition and standardization of silymarin

Silymarin is a multicomponent natural product rather than a single active molecule and contains several flavonolignans together with related polyphenolic constituents (Křen and Valentová, 2022; Bijak, 2017). Analysis of over 40 commercial milk-thistle extracts revealed significant stability in the ratios of selected flavonolignans (Chen et al., 2024), while overall compositional surveys suggest that the combination of flavonolignan content may fluctuate depending on the processing methods, plant chemotypes and plant material used (Chambers et al., 2017). Its major constituents include silybin (silibinin), isosilybin, silychristin, and silydianin and minor constituents include taxifolin along with other minor compounds in the chemical profile (Křen and Valentová, 2022; Bijak, 2017). Silybin is usually considered as the principal bioactive compound, but variations in concentrations of the other compounds, as well as their biotransformation, can alter the properties of the whole extract (Bijak, 2017; Valentová et al., 2020).

This heterogeneity directly impacts the way the antifibrotic literature should be read (Chen et al., 2024; Chambers et al., 2017). Crude or standardized silymarin extracts, purified silibinin, phospholipid complexes, nanocarriers and formulations as multi-components in dietary supplements have been used in investigations (Chambers et al., 2017; Barzaghi et al., 1990). The compositions and formulations affect the exposure and the product-level behavior and thus the results of these preparations are not assumed to be pharmacologically interchangeable (Chambers et al., 2017; Li et al., 2018).

Therefore, chemical standardization should be regarded as a part of the mechanistic rigor and not just a manufacturing detail. Future reports should at least include the plant source, methods of extraction and production if available, the levels of the major flavonolignans, and the percentage of silybin A/B, if available, and should be chromatographically or chemically characterized (Chen et al., 2024; Chambers et al., 2017). This information is critical when mechanistic studies are translated to clinical studies, since product heterogeneity and exposure (dependent on the formulation) may mask actual efficacy signals (Chambers et al., 2017; Li et al., 2018).

The major flavonolignans share a related flavonoid-phenylpropanoid scaffold, but silybin A/B, isosilybin A/B, silychristin, and silydianin differ in stereochemistry and regiochemistry (Křen and Valentová, 2022; Bijak, 2017). These differences may affect membrane interactions, enzyme and transporter recognition, biotransformation, redox behavior, and ultimately systemic exposure. Stereoselective pharmacokinetics have indeed been reported for the major flavonolignans (Zhu et al., 2013; Bijak, 2017), and silybin A and B show different systemic exposures. However, no clear structure-antifibrotic activity relationship has been established. Most constituent-level comparisons concern hepatoprotective, antioxidant, antiproliferative, metabolic, or pharmacokinetic endpoints rather than reversal of established fibrosis. Structural variation should therefore be viewed as a source of pharmacological heterogeneity, not as evidence that one flavonolignan has intrinsically greater antifibrotic potency than another.

The principal constituents and formulation categories relevant to antifibrotic pharmacology are summarized in Table 1.

**TABLE 1:** Key constituents and formulation categories relevant to the antifibrotic pharmacology of silymarin.

Constituent or formulation	Pharmacological significance	Translational consideration
Silymarin extract	Multicomponent flavonolignan preparation with reported antioxidant, anti-inflammatory, and antifibrotic activity (Křen and Valentová, 2022; Bijak, 2017). Overall composition varies among preparations (Chambers et al., 2017), although selected flavonolignan ratios are relatively stable (Chen et al., 2024)	Processing, plant chemotype, and source can alter product composition (Chambers et al., 2017), which may complicate reproducibility and comparison across pharmacological studies (Chambers et al., 2017; Li et al., 2018)
Silybin/silibinin	Principal bioactive constituent. Silibinin reduces hepatic stellate-cell proliferation and migration (Ezhilarasan et al., 2016); in cultured skin fibroblasts, it shifts matrix regulation toward lower type I collagen production with reduced Smad2/3 activation (Cho et al., 2013)	Where relevant, studies should report silybin A/B composition and formulation characteristics (Chen et al., 2024; Bijak, 2017). Better component-level standardization improves cross-study comparability (Chambers et al., 2017)
Isosilybin, silychristin, silydianin	These constituents contribute to the overall flavonolignan profile and may modify the behavior of the multicomponent extract (Křen and Valentová, 2022; Bijak, 2017)	They are frequently under-characterized. More complete compositional reporting would improve reproducibility (Chen et al., 2024; Chambers et al., 2017)
Silybin-phosphatidylcholine complex	Phosphatidylcholine complexation increases systemic silybin exposure relative to conventional preparations (Barzaghi et al., 1990; Yanyu et al., 2006); oily-medium soft-gel products can also enhance bioavailability (Méndez-Sánchez et al., 2019)	Potentially useful for exposure-guided development, but formulation differences must be considered when clinical results are compared (Barzaghi et al., 1990; Méndez-Sánchez et al., 2019)
Micellar, liposomal, nanocolloid, redox-nanoparticle, or sustained-release systems	Micellar silymarin increases systemic exposure in humans (Chang et al., 2025). Experimental nanoliposomes, modified liposomes, liver-targeted nanocolloids, PPC carriers, redox nanoparticles, and sustained-release matrices alter release, delivery, or pharmacological activity (Lu et al., 2007; Chen et al., 2021; Wu et al., 2025; Wen et al., 2025; Li Y. et al., 2025; Nguyen et al., 2026)	Each platform requires formulation-specific evaluation of safety, pharmacokinetics, manufacturability, and efficacy. Increased exposure or preclinical targeting does not by itself establish clinical antifibrotic benefit (Chang et al., 2025; Wu et al., 2025; Li Y. et al., 2025)

### 3.2 Pharmacokinetic limitations and formulation-dependent translational relevance

One of the main problems associated with the use of conventional silymarin is limited and unstable oral exposure to obtain a reproducible therapeutic action (Zhu et al., 2013; Barzaghi et al., 1990). Systemic exposure to silybin in humans and experimental animals can be significantly modified by changes in formulation, such as complexation with phosphatidylcholine (Barzaghi et al., 1990; Li et al., 2018; Méndez-Sánchez et al., 2019; Yanyu et al., 2006). The extent of this effect is demonstrated in a crossover study in 16 healthy subjects in which the micellar preparation resulted in a peak exposure ∼19 fold and a systemic exposure over 24 h ∼11 fold higher than that obtained with the unformulated comparator (Chang et al., 2025). These differences create a pharmacokinetic advantage, rather than antifibrotic efficacy.

The pharmacokinetic properties of each of the individual flavonolignans found in silymarin are quite distinct; therefore, it is difficult to consider silymarin as a single pharmacokinetic entity. Six primary flavonolignans were detected in the blood of healthy volunteers who received a standardized milk-thistle extract; however, there was considerable variability in the exposures to the free form of each of these compounds: silybin A > silybin B > isosilybin B > isosilybin A > silychristin > silydianin (Zhu et al., 2013). Following the administration of a single dose of 175–525 mg, the average concentration (Cmax) of free silybin A ranged from approximately 107–299 ng/mL while the average concentration (Cmax) of free silybin B ranged from approximately 31–121 ng/mL. Following the administration of 175 mg three times daily until a steady-state was reached, the average concentrations (Cmax) of free silybin A, free silybin B and free isosilybin B were approximately 135 ng/mL, 42 ng/mL and 27 ng/mL, respectively (Zhu et al., 2013). In addition to being stereoselective, there are several pathways involved in the disposition of silymarin components. One such pathway involves the formation of glucuronides and sulfates through Phase II metabolism. These reactions result in only a very small percentage of the total amount of silymarin components being present as free compound in the bloodstream (Zhu et al., 2013; Bijak, 2017; Li et al., 2018). Additionally, biliary excretion and enterohepatic circulation of conjugated metabolites play a major role in the disposition of silymarin components. The above differences in disposition are important when conducting mechanistic studies using purified silybin or silibinin, as you cannot assume that the exposure of the purified compound represents the exposure of the multi-component extract.

The exposure is particularly relevant in fibrosis as the matrix remodeling occurs over an extended period of time and does not reflect a transient biochemical reaction (Iredale et al., 1998; Rockey and Friedman, 2021). There must be concerted changes in ECM production and degradation, as well as alterations in the activation states of fibrogenic cells, for meaningful regression to occur (Iredale et al., 1998; Rockey and Friedman, 2021). This means that prolonged tissue levels might be required to affect these processes. Modest or negative results with conventional formulations cannot automatically rule out potential antifibrotic activity, but they certainly point to the need for exposure-response analyses, pharmacokinetically supported dosing, and trial designs controlled for formulation (Zhu et al., 2013; Kheong et al., 2017).

To overcome these constraints, a variety of delivery strategies have been considered. Phospholipid complexes and micellar systems are mainly designed to enhance the systemic exposure (Barzaghi et al., 1990; Chang et al., 2025) whilst sustained release and nanoliposomal systems can be used to manipulate the release kinetics or the tissue level pharmacology (Lu et al., 2007; Chen et al., 2021). Newly emerging carriers with a focus on fibrosis can be classified into two overall approaches. Platforms which focus particularly on improving hepatic access or contribution of oral exposure to the liver include HA-DOCA nanocolloids and silica-containing redox nanoparticles (Wen et al., 2025; Nguyen et al., 2026), and platforms directed at altering the fibrotic microenvironment or improving HSC/ECM targeting include catalase-modified liposomes and a dual-targeting PPC liposomal system (Wu et al., 2025; Li Y. et al., 2025). It is important to note that, collectively, these studies demonstrate that formulation can significantly impact preclinical pharmacology, without proof of clinical antifibrotic efficacy in humans. When local administration is used, it may be another option for increasing tissue exposure and decreasing reliance on systemic uptake in the event of a localized fibro-inflammatory disorder.

### 3.3 Antifibrotic pharmacological mechanisms

For clarity, we distinguish three categories of fibrosis-related evidence. “Antifibrogenic” effects refer to prevention or attenuation of matrix accumulation while an injurious stimulus is ongoing. “Effects on established fibrosis” refer to treatment initiated after substantial matrix deposition is already present. “Fibrosis regression” or “fibrolysis” requires evidence that pre-existing scar has been removed or remodeled, ideally after the initiating injury has ceased. Changes in oxidative stress, inflammation, tissue injury, fibroblast or HSC activation, and collagen-related markers may be relevant to fibrogenesis, but none alone proves regression of established fibrosis. These distinctions are applied throughout the mechanistic, preclinical, and clinical sections.

#### 3.3.1 Modulation of TGF-β/Smad signaling

TGF-β/Smad signaling is a fundamental axis of profibrotic activity that regulates activation and differentiation of fibroblasts and their collagen production. In experiments in peritoneal and hepatic fibrosis, silymarin-containing interventions have been shown to inhibit this signalling network (Bai et al., 2023; Eraky et al., 2018). In thioacetamide-induced hepatic fibrosis, a comparison of the effects of silymarin, caffeine, and their combination revealed a decrease in TGF-β1-, CTGF-, α-SMA-, and LPAR1-related markers, especially in the combination-treated group (Eraky et al., 2018). Silymarin also decreased CTGF, hepatic hydroxyproline and fibrosis in the liver on histopathological examination in CCl4-treated rats (Tzeng et al., 2013). Similar trends were observed in renal models: nanoliposomal silymarin co-suppressed TGF-β/Smad and JAK2/STAT3/SOCS1 signaling in diabetic nephropathy (Chen et al., 2021); and oral silymarin in a 2024 thioacetamide model resulted in renal functional improvement, reduced interstitial fibrosis, collagen I, CTGF and TGF-β/Smad-related abnormalities (Mekawy et al., 2024).

Overall, these findings indicate that silymarin-related interventions can modulate canonical profibrotic signaling, although a single uniform proximal mechanism cannot be inferred. Different disease models, constituents and formulations indicated different apparent points of activity and levels of activity (Chen et al., 2021; Bai et al., 2023; Mekawy et al., 2024). Future mechanistic studies should distinguish secondary effects resulting from decreased tissue damage from the direct inhibition of TGF-β/Smad activity at the fibroblast level, and should include pathway-specific perturbation or rescue experiments wherever possible.

#### 3.3.2 Oxidative stress and cytoprotective responses

Redox imbalance is a recurrent feature of tissue injury that can amplify fibrogenic signaling. Silymarin has shown cytoprotective and antioxidant activity in doxorubicin- and ethanol-induced hepatic injury (Patel et al., 2010; Song et al., 2006). In experimental NASH, silibinin reduced oxidative damage alongside steatosis and fibrosis-associated changes (Ou et al., 2018), and oral silymarin improved several injury-related features in a juvenile murine NASH model (Marin et al., 2017).

These redox effects are relevant to MASLD/MASH, in which oxidative injury intersects with metabolic dysfunction, inflammatory signaling, and activation of fibrogenic cells (Ou et al., 2018; Marin et al., 2017). In diabetic renal injury, oxidative stress also converges with TGF-β/Smad and JAK2/STAT3/SOCS1 pathways (Chen et al., 2021). Silibinin has additionally been associated with improvement of both hepatic and myocardial injury in NASH mice (Salamone et al., 2012). Nevertheless, describing silymarin simply as an antioxidant would be reductive: the experimental literature also implicates inflammatory, apoptotic, metabolic, and matrix-remodeling processes (Patel et al., 2010; Ou et al., 2018).

#### 3.3.3 Suppression of inflammatory amplification

Inflammatory signaling helps sustain fibrosis by perpetuating tissue damage and maintaining activation of fibrogenic effector cells. In a murine NASH model, silybin reduced inflammatory signaling in parallel with oxidative injury and fibrosis-related changes (Ou et al., 2018). An irinotecan-induced NASH model showed a non-linear dose response to silymarin: low and intermediate doses improved several inflammatory and fibrogenic indices, whereas the highest tested dose was less favorable (Assis-Júnior et al., 2017). CCl4-induced hepatic fibrosis was also accompanied by silymarin-associated changes in mast cells and TGF-β1 (Jeong et al., 2005). At the cellular level, silymarin suppressed TNF-triggered NF-κB and c-Jun N-terminal kinase activation (Manna et al., 1999), and in LPS-stimulated macrophages it limited inflammation-related morphological changes through NF-κB inhibition (Kim et al., 2015).

These findings support the view that silymarin-related compounds can influence the interface between inflammatory amplification and fibrogenesis (Ou et al., 2018; Jeong et al., 2005). Direct pathway evidence for NF-κB and related signaling, however, remains predominantly preclinical (Manna et al., 1999; Kim et al., 2015). It is therefore more accurate to describe silymarin as a potential modulator of fibro-inflammatory networks than as either a purely hepatoprotective agent or a direct inhibitor of collagen synthesis.

#### 3.3.4 Fibroblast activation, myofibroblast differentiation, and ECM remodeling

Fibrosis is determined not only by excessive matrix synthesis but also by the failure to resolve deposited ECM (Iredale et al., 1998; Rockey and Friedman, 2021). Regression therefore depends on shifts in cellular phenotype and matrix-degrading activity as well as on reduced collagen production (Iredale et al., 1998; Rockey and Friedman, 2021). In cultured human skin fibroblasts, silibinin shifted matrix regulation toward a less collagen-producing phenotype, with lower type I collagen expression and reduced Smad2/3 activation (Cho et al., 2013). Hepatic stellate-cell studies likewise report reduced activation, proliferation, and migration after exposure to silymarin or silibinin (Fuchs et al., 1997; Ezhilarasan et al., 2016), and animal liver-fibrosis models show reductions in collagen-associated mediators such as CTGF (Tzeng et al., 2013). Evidence for reversal of established fibrosis is less consistent. In one CCl4-withdrawal model, neither silymarin nor silibinin produced a significant fibrolitic effect (Muriel et al., 2005), whereas a later high-dose silymarin study reported greater histological restoration and lower α-SMA than in untreated controls (Tsai et al., 2008). Thus, prevention or attenuation of fibrogenesis should not be equated with proven reversal of established scar.

Whether fibrosis truly regresses is likely to depend on the balance among collagen turnover, MMP activity, TIMP-mediated inhibition, and survival or deactivation of fibrogenic cells (Iredale et al., 1998; Rockey and Friedman, 2021). The divergent CCl4-withdrawal studies (Muriel et al., 2005; Tsai et al., 2008) illustrate why suppression of ongoing injury must be distinguished from active remodeling of established scar. Future experiments should therefore incorporate matrix-centered outcomes such as quantitative collagen measurements, α-SMA, MMP/TIMP balance, hydroxyproline, and validated histological or imaging measures of fibrosis.

#### 3.3.5 Emerging mechanistic nodes: metabolic crosstalk, ferroptosis, and direct target engagement

Recent studies have extended the mechanistic picture beyond the better-known TGF-β/Smad and NF-κB pathways. These findings need to be interpreted according to the specific intervention that was tested rather than treated as evidence for a uniform “silymarin class” effect. In a 2025 MASH model, purified silybin altered metabolic crosstalk between hepatocytes and stellate cells: it improved the mitochondrial lipid environment and complex II activity, reduced succinate accumulation and release, and made conditioned medium less stimulatory to LX-2 cells (Yang et al., 2025). These findings support an upstream metabolic mechanism for silybin in that model, but they should not be extrapolated directly to multicomponent silymarin preparations.

The studies on kidney and lungs offer more specific target-level evidence, but they vary on their degree of validation. For example, in renal fibrosis, the FTH1 study tested silybin; the researchers found a silybin-FTH1 interaction and performed both loss- and gain-of-function experiments. They demonstrated that when FTH1 was deleted, ferroptotic damage and fibrosis were increased, whereas when FTH1 was overexpressed, it offered protection against these effects (Hu et al., 2025). This demonstrates a clear case for target-dependent efficacy, yet independent validation and direct *in vivo* demonstrations of target engagement remain to be done. Similarly, in pulmonary fibrosis, silibinin was shown to physically interact with ANXA6 and to modify ER/oxidative stress and PI3K-AKT-mTOR-related responses (Guan et al., 2026). However, simply binding to a molecule does not prove that ANXA6 engagement is either required or sufficient for the overall antifibrotic phenotype. Thus, generally speaking, target identification, physical interaction with the target, target engagement in the correct tissue, and target-dependent reversal of a disease state provide different types of causal evidence and therefore require distinct descriptions.

Whether the proposed mechanisms can be achieved at a clinically relevant dose remains unclear. Under typical experimental dosing conditions with the use of standard oral silymarin, the concentration of free silybin in humans is typically less than 1 μM (Zhu et al., 2013); however, the use of different formulations may lead to higher levels of exposure (Barzaghi et al., 1990; Chang et al., 2025). At present, there are no published reports that have allowed for an accurate assessment of the relationship between the experimental concentrations of succinate, FTH1/ferroptosis and ANXA6 (Yang et al., 2025; Hu et al., 2025; Guan et al., 2026) and the actual concentration achieved in human target tissues. There are no comparisons possible among the studies as to how plasma or tissue concentrations were determined. Furthermore, the extent to which human target tissues were exposed is unknown. Therefore, it appears that the mechanism(s) described above should be considered as potential preclinical findings, as opposed to clinically valid mechanisms that can be demonstrated in humans at clinically relevant exposures. Additional translational research would be required to establish a correlation between the dose/concentration-response relationships, determine the amount of unbound drug available for distribution throughout the body and demonstrate that the mechanism of action occurs at clinically achievable doses.

The network-level pharmacological mechanisms relevant to the antifibrotic potential of silymarin are summarized in Figure 1.

**FIGURE 1:**
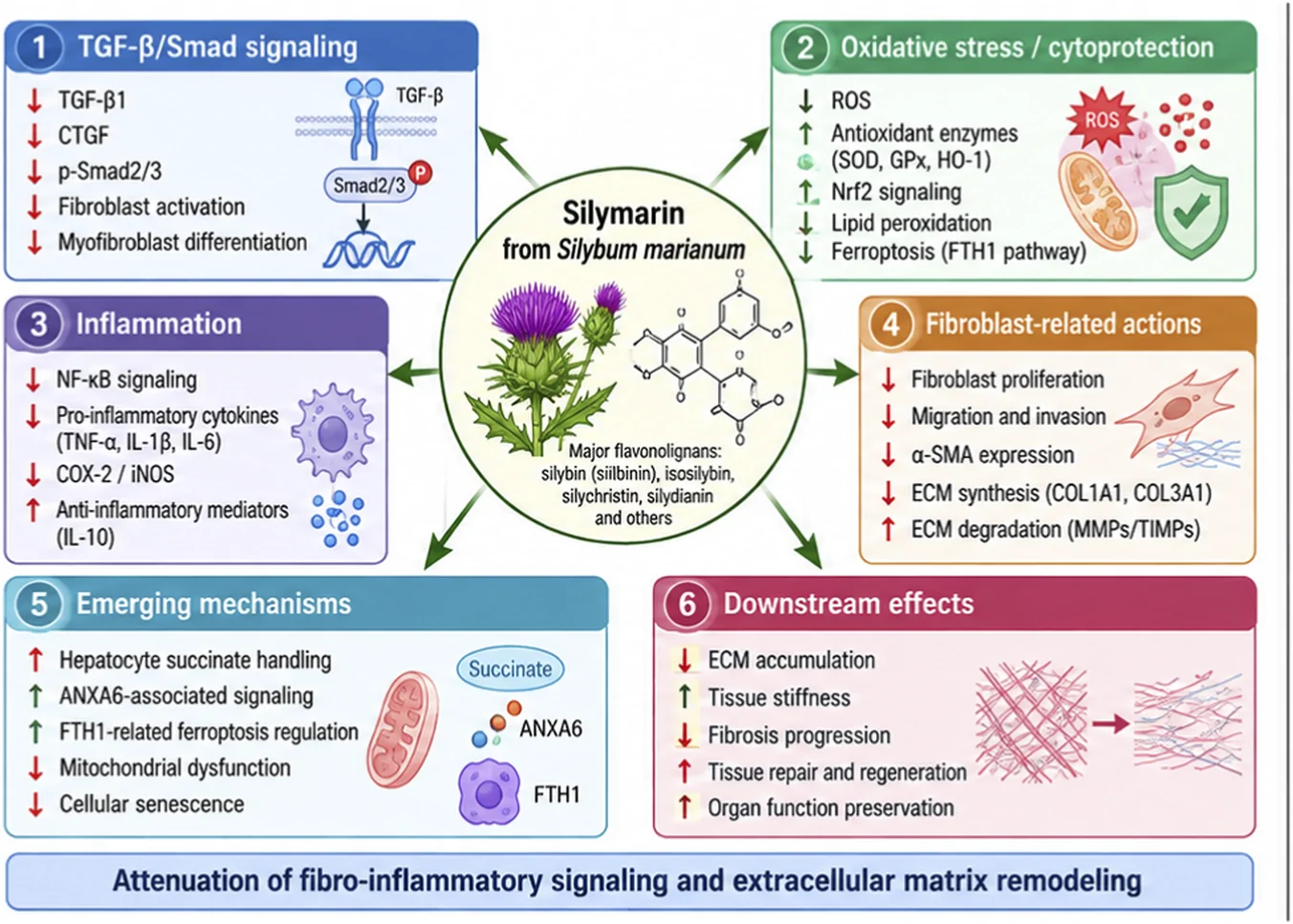
Network-level pharmacology relevant to silymarin-related fibro-inflammatory effects. The strength of evidence differs across the mechanisms shown. TGF-β/Smad signaling, oxidative stress, inflammatory signaling, fibrogenic-cell activation, and ECM remodeling are supported by multiple preclinical studies, whereas succinate signaling, FTH1-related ferroptosis, and ANXA6-associated signaling remain emerging, constituent-specific mechanisms. These findings should not be interpreted as evidence that every altered pathway represents a direct molecular target. Evidence for regression of established fibrosis also remains inconsistent.

### 3.4 Preclinical evidence across fibro-inflammatory models

Preclinical studies continue to dominate the evidence base for the antifibrotic effects of silymarin and silibinin. In liver models, silymarin and silibinin have been associated with reduced stellate-cell activation, collagen-related mediators, inflammatory injury, and TGF-β-related signaling (Fuchs et al., 1997; Eraky et al., 2018; Tzeng et al., 2013). A few dietary steatohepatitis models also provide evidence for the reduction of oxidative damage, inflammatory signaling, and fibrosis associated changes (Ou et al., 2018; Marin et al., 2017). The activity of silymarin outside the liver is shown by suppression of TGF-β/Smad associated remodeling in experimental peritoneal fibrosis (Bai et al., 2023) and by nanoliposomal silymarin in diabetic nephropathy (Chen et al., 2021) and by oral silymarin in reduction of interstitial fibrosis and TGF-β/SMAD associated abnormalities in thioacetamide induced renal injury (Mekawy et al., 2024). Silibinin also lowered the level of type-I collagen in human skin fibroblasts (Cho et al., 2013). In contrast, specific tests of regression following CCl4 withdrawal have yielded conflicting results (Muriel et al., 2005; Tsai et al., 2008).

Across organ systems, a similar pattern emerges. Silybin has been linked to hepatocyte-stellate-cell metabolic crosstalk and FTH1-dependent regulation of ferroptosis, whereas silibinin has been linked to ANXA6-associated stress signaling in lung fibrosis models (Yang et al., 2025; Hu et al., 2025; Guan et al., 2026). Engineered formulations have separately been used to modify exposure or tissue distribution in experimental hepatic fibrosis (Wu et al., 2025; Wen et al., 2025; Li Y. et al., 2025; Nguyen et al., 2026). Taken together, these studies show that silymarin-related interventions can influence several interconnected biological pathways, but they do not establish classical polypharmacology or multiple independently validated direct targets. Some pathway changes may arise secondarily from reduced tissue injury or from modulation of a smaller number of proximal targets. Accordingly, we use “multitarget pharmacology” here as a network-level concept; whether this breadth of activity provides a therapeutic advantage in multifactorial disease remains a hypothesis.

Representative preclinical evidence across fibro-inflammatory models is summarized in Table 2.

**TABLE 2:** Representative preclinical evidence for antifibrotic and fibro-inflammatory effects of silymarin or silibinin, including 2025–2026 mechanistic studies.

Model or setting and evidence stage	Intervention	Principal fibrosis-related observations	Representative references
Mechanistic/cellular (fibrosis stage not applicable) Hepatic stellate cells	Silymarin or silibinin	Lower stellate-cell activation, proliferation, and migration	(Fuchs et al., 1997; Ezhilarasan et al., 2016)
Prevention/attenuation during ongoing injury Experimental liver fibrosis	Silymarin (Tzeng et al., 2013); silymarin, caffeine, or their combination (Eraky et al., 2018)	Reduced TGF-β1/CTGF-related signaling, α-SMA, hydroxyproline, collagen accumulation, and fibrosis; effects of the combination regimen in Eraky et al. (2018) should not be ascribed to silymarin alone	(Eraky et al., 2018; Tzeng et al., 2013)
Established fibrosis; regression after injury withdrawal Established CCl4-induced liver fibrosis after toxin withdrawal	Silymarin or silibinin	Discordant results: one study found no significant fibrolitic effect, whereas another reported greater histological recovery and lower α-SMA with high-dose silymarin	Muriel et al., 2005; Tsai et al., 2008)
Prevention/attenuation in metabolic injury Dietary or metabolic steatohepatitis models	Silymarin or silibinin	Attenuation of oxidative injury, inflammatory signaling, steatosis, and fibrosis-associated changes	(Ou et al., 2018; Marin et al., 2017)
Prevention/attenuation during ongoing injury Peritoneal fibrosis	Silymarin	Reduced TGF-β/Smad signaling and fibro-inflammatory remodeling	(Bai et al., 2023)
Prevention/attenuation during ongoing injury Diabetic nephropathy model	Nanoliposomal silymarin	Reduced renal injury with concurrent suppression of TGF-β/Smad and JAK2/STAT3/SOCS1 signaling	(Chen et al., 2021)
Treatment/attenuation during ongoing injury Thioacetamide-induced renal fibrosis	Silymarin	Improved renal dysfunction and interstitial fibrosis with reductions in collagen I, CTGF, and TGF-β/SMAD-related abnormalities	(Mekawy et al., 2024)
Mechanistic/cellular (fibrosis stage not applicable) Skin fibroblast model	Silibinin	Shift toward a less collagen-producing phenotype, with lower type I collagen expression and reduced Smad2/3 activation	(Cho et al., 2013)
Prevention/attenuation of a fibrotic response Smoke-related fibrotic response	Silibinin	Attenuation of TGF-β1/Smad2/3-associated fibrotic responses	(Ko et al., 2017)
Prevention/attenuation in metabolic injury MASH-associated liver fibrosis (CDAHFD)	Silybin	Improved hepatocyte mitochondrial/complex II function with less extracellular succinate signaling and weaker stellate-cell activation	(Yang et al., 2025)
Treatment/attenuation; regression not established Folic acid-induced renal fibrosis	Silybin	FTH1-associated improvement in iron handling and ferroptotic injury, accompanied by less renal fibrotic remodeling	(Hu et al., 2025)
Fibrosis-treatment model; regression not established Bleomycin pulmonary fibrosis and human lung fibroblasts	Silibinin	ANXA6-associated reduction in PI3K-AKT-mTOR activity, cellular stress, and acquisition of a matrix-producing fibroblast phenotype	(Guan et al., 2026)
Prevention/attenuation with engineered delivery CCl4-induced liver fibrosis with oral nanocarrier	Silymarin-loaded silica redox nanoparticles	Greater hepatic exposure with lower collagen deposition and fibrogenic-marker expression than free silymarin	(Nguyen et al., 2026)
Prevention/attenuation with engineered delivery CCl4-induced liver fibrosis with redox-responsive liposomes	Catalase-modified silymarin liposomes	Lower oxidative injury and fibrosis-associated biochemical markers in the experimental model	(Wu et al., 2025)
Prevention/attenuation with engineered delivery CCl4-induced liver fibrosis with oral liver-targeted nanocolloid	Silybin-loaded HA-DOCA zein nanocolloids	Improved oral hepatic delivery and stronger fibrosis-related efficacy than free silybin	(Wen et al., 2025)
Prevention/attenuation with engineered delivery CCl4-induced liver fibrosis with HSC/ECM dual-targeting carrier	Silybin B-retinoic-acid/PPC liposomal platform with collagenase I	Improved fibrotic-barrier penetration and HSC/ECM targeting, with lower collagen burden	(Li et al., 2025b)

### 3.5 Clinical evidence and translational limitations

The experimental literature is much more consistent than human evidence (Fried et al., 2012; Kheong et al., 2017). An early randomized cirrhosis trial showed a survival benefit, with more apparent benefits in alcoholic cirrhosis and Child A disease (Ferenci et al., 1989), but later in the same disease in a multicenter randomized trial, no survival benefit was seen (Parés et al., 1998). Randomized treatment of chronic hepatitis C (CHC) with silymarin was well tolerated without any significant beneficial HCV RNA or aminotransferase results (Fried et al., 2012). Observational data collected from HALT-C were more subtle, showing that the self-reported use of silymarin was correlated with less histological progression from fibrosis to cirrhosis, but not with clinical outcomes (Freedman et al., 2011). The combined results of these studies do not provide evidence for a clinically significant disease modifying impact in chronic hepatitis C.

A similar distinction should be made in relation to clinical literature as well. The majority of published clinical studies did not examine if there was a decrease in the amount of fibrotic tissue present in the liver or whether the existing fibrosis had undergone histological regression. Rather than using direct measures of fibrotic change, many used biochemical, virological, renal, survival, or other non-invasive surrogate markers as their study endpoints. Therefore, both positive and negative findings require interpretation with respect to the type of product administered, the degree of exposure expected to have occurred, the initial severity of the disease, the length of therapy provided, and the extent to which the chosen study endpoint reflects fibrotic changes.

Randomized data for NASH/MASLD are also mixed. In biopsy-proven NASH, there was not a significant effect of silymarin on the prespecified primary outcome defined by NAFLD activity score, but a signal of fibrosis improvement was seen (Kheong et al., 2017). The SyNCH trial also showed safety to be acceptable with higher than normal doses, and showed no significant improvement in its main histological endpoint (Navarro et al., 2019). In a 24-week randomized trial of MASLD, participants had an improvement in liver-stiffness measurements (Jin et al., 2024). This is better regarded as a non-invasive signal, rather than as a demonstration of scar regression, in the absence of paired histology. A 2025 Cochrane Review that included 17 trials and 2069 people concluded that overall risks and benefits were unclear, as the evidence included was low and very low certainty and was clinically heterogeneous (Wang et al., 2025).

In addition, the metabolic context of MASLD/MASH is important. Insulin resistance, mitochondrial stress, inflammation, and the two-way interaction between the liver and diabetes are all aspects of the disease process (Manshi and Koiri, 2026) that have an impact on the rate at which fibrotic tissue forms as well as how a patient appears to respond to treatment. Thus, fibrotic outcome cannot be evaluated independently of the patient’s underlying metabolic phenotype. There have been several recent hypotheses suggesting that metabolic flexibility impairment and/or altered circadian/bioenergetic timing could account for some of the variation in MASLD (Dash and Koiri, 2026). However, these are currently hypothesis-generating only and should not be used as factors in determining whether a patient will respond positively to silymarin.

Clinical findings in renal disease are similarly inconsistent. In diabetic nephropathy, a 3-month trial of silymarin added to renin-angiotensin system inhibitors reported reductions in albuminuria and selected inflammatory and oxidative-stress markers (Fallahzadeh et al., 2012); however, the markers measured represent only surrogate markers and are not sufficient to prove reversal of structural renal fibrosis. In a subsequent randomized controlled trial of 102 normotensive patients with type 2 diabetes and proteinuria, however, an additional silymarin treatment was not found to significantly affect the primary composite outcome, renal outcomes, or proteinuria (Voroneanu et al., 2017). Available human data thus do not allow for a categorical ‘no’ or for therapeutic overstatement. While generally well tolerated in trials, data on antifibrotic efficacy are very context, formulation and end-point dependent (Fried et al., 2012; Kheong et al., 2017). Future clinical studies should focus on validated fibrosis as a primary endpoint instead of relying primarily on biochemical measures of disease activity (Navarro et al., 2019; Jin et al., 2024; Wang et al., 2025) and should involve a pharmacokinetic rationale for dose and formulation.

Representative clinical studies and their fibrosis-related outcomes are summarized in Table 3.

**TABLE 3:** Summary of clinical studies evaluating silymarin in fibrosis-related or fibro-inflammatory conditions.

Study	Condition/design and baseline fibrosis	Preparation/formulation, dose, and duration	Fibrosis-related or disease-modifying endpoint	Interpretation and translational limitation
(Fried et al., 2012)	Chronic hepatitis C; RCT, n = 154; chronic HCV with elevated ALT; baseline fibrosis was heterogeneous because liver biopsy was not required	Standardized Legalon silymarin dry extract; 420 mg or 700 mg three times daily vs. placebo for 24 weeks	Primary endpoint: ALT response; secondary endpoints: HCV RNA and quality of life; no fibrosis-specific structural endpoint	No significant improvement in ALT or viral load; therefore, this was not a direct test of fibrosis regression
(Kheong et al., 2017)	NASH; RCT, n = 99; biopsy-proven NASH with paired baseline and follow-up histology	Oral silymarin 700 mg three times daily vs. placebo for 48 weeks; detailed flavonolignan standardization is relevant when comparing this trial with others	Primary endpoint: ≥30% reduction in NAS; secondary measures included fibrosis stage, liver stiffness, and metabolic/liver outcomes	Primary NAS endpoint negative; a possible fibrosis-related signal was observed; the fibrosis finding was secondary and requires confirmation
(Navarro et al., 2019)	Non-cirrhotic NASH; RCT, n = 78; with histological assessment	High-dose silymarin; 48 weeks; formulation details and achieved exposure should be considered in cross-trial comparisons	Histology; fibrosis was assessed histologically rather than through biochemical surrogates	Acceptable safety, but no significant effect on the primary histological endpoint
(Jin et al., 2024)	MASLD; RCT, n = 83; baseline fibrosis was assessed non-invasively rather than by paired biopsy	Oral silymarin 103.2 mg/day vs. placebo for 24 weeks	FibroScan liver stiffness, steatosis, biochemical measures, and gut-microbiota outcomes; no paired histology	Liver-stiffness measurements decreased; without paired histology, this remains a non-invasive fibrosis-related signal rather than proof of scar regression
(Fallahzadeh et al., 2012)	Diabetic nephropathy; RCT, n = 60; structural renal fibrosis was not measured directly	Silymarin plus renin-angiotensin system inhibitor; 3 months; oral preparation; constituent-specific exposure was not reported in the summarized evidence	Proteinuria and inflammatory/oxidative markers; endpoints were renal and inflammatory surrogates rather than measures of fibrosis regression	Proteinuria and urinary TNF-α/MDA decreased
(Voroneanu et al., 2017)	T2DM with proteinuria; randomized, n = 102; renal fibrosis stage was not structurally defined	Silymarin + optimized RAS blockade vs. placebo; 2 years; oral silymarin adjunct; detailed constituent-level standardization and exposure were not available in the summarized evidence	Primary composite renal/clinical outcome; eGFR; proteinuria	No significant benefit for the primary composite endpoint, renal outcomes, or proteinuria
(Ferenci et al., 1989)	Cirrhosis; RCT, n = 170; established cirrhosis; survival, not direct fibrosis remodeling, was assessed	Silymarin vs. placebo; 2 years; historical trial with limited constituent-level pharmacokinetic and standardization data by current standards	Survival	Higher 4-year survival was reported overall, with stronger signals in alcoholic cirrhosis and Child A disease
(Parés et al., 1998)	Alcoholic cirrhosis; RCT, n = 200; alcoholic cirrhosis confirmed histologically or by laparoscopy	Oral silymarin 450 mg/day (150 mg three times daily) vs. placebo for 2 years	Primary endpoint: survival; secondary endpoint: progression of liver failure; no direct measure of fibrosis regression	No survival advantage

## 4 Discussion

### 4.1 Hypothesis-generating future application: mucosal fibro-inflammatory remodeling

CRSwNP is included here only as a hypothesis-generating future application, not as part of the current antifibrotic evidence base. Type 2 inflammation, epithelial-barrier dysfunction, and ECM remodeling contribute to disease persistence and recurrence (Bachert et al., 2018; Fokkens et al., 2020; Du et al., 2021; Vaitkus et al., 2025), and several of these processes overlap with pathways modulated by silymarin-related interventions in other tissues.

No published evidence currently supports silymarin as a treatment for CRSwNP. Any future work should begin with validated nasal-polyp fibroblast or epithelial-stromal models and exposure-appropriate local-delivery studies before clinical translation is considered.

### 4.2 Overall synthesis and conclusion

Silymarin should be considered a chemically heterogeneous natural product with a broad but incompletely defined fibro-inflammatory pharmacology. Its flavonolignan constituents, particularly silybin-related compounds, are well characterized chemically (Křen and Valentová, 2022; Bijak, 2017), yet product-level composition can vary despite the relative stability of some characteristic flavonolignan ratios (Chen et al., 2024; Chambers et al., 2017). Preclinical studies implicate TGF-β-related signaling, redox and NF-κB-associated inflammatory pathways, fibroblast behavior, and ECM regulation (Bai et al., 2023; Ou et al., 2018; Cho et al., 2013), with newer work adding hepatocyte succinate metabolism, FTH1-dependent ferroptosis, and ANXA6-linked stress signaling (Yang et al., 2025; Hu et al., 2025; Guan et al., 2026). These data support antifibrotic potential across liver, kidney, lung, peritoneal, and skin-related models, but evidence for active reversal of established fibrosis remains inconsistent (Muriel et al., 2005; Tsai et al., 2008). Delivery studies further show that formulation can materially alter tissue exposure and experimental efficacy (Wu et al., 2025; Wen et al., 2025; Li Y. et al., 2025; Nguyen et al., 2026). Human studies are much less persuasive: randomized NASH trials have yielded negative or modest primary results (Kheong et al., 2017; Navarro et al., 2019), the overall MASLD evidence remains uncertain according to a 2025 Cochrane review (Wang et al., 2025), and the reported reduction in liver stiffness in a 2024 MASLD trial was not accompanied by proof of histological regression (Jin et al., 2024). The translational gap is therefore likely multifactorial, involving product heterogeneity, limited oral exposure, incomplete standardization, and suboptimal endpoint selection (Zhu et al., 2013; Chambers et al., 2017). Future development should focus on chemically defined preparations, exposure-informed dosing, optimized delivery, validated target engagement, and fibrosis-specific outcomes. On current evidence, silymarin remains an investigational adjunctive candidate rather than an established antifibrotic treatment. Viewed through this framework, each study can be assessed by asking what intervention was tested, what exposure was likely to be achieved, how strong the mechanistic evidence was, at what stage of fibrosis treatment began, and whether the endpoint directly measured fibrosis. On this hierarchy, the evidence is strongest for prevention or attenuation of fibro-inflammatory processes in preclinical models, less consistent for effects on established fibrosis, and currently insufficient to support a general claim that silymarin or one of its components reproducibly reverses fibrosis in humans.

### 4.3 Future perspectives

Future development should begin with rigorous definition and control of the intervention being tested. The conventional silymarin extracts, purified form of silibinin, phospholipid complexes, nanocarriers and other standardized preparations should be clearly differentiated in studies (Chen et al., 2024; Chambers et al., 2017). Some characteristic flavonolignan ratios do seem to be relatively constant from extract to extract (Chen et al., 2024), but there is still product-to-product variation (Chambers et al., 2017; Li et al., 2018). Chemical characterisation should be also linked to the pharmacological findings by providing the major flavonolignan content, silybin A/B composition and batch consistency (Chen et al., 2024; Chambers et al., 2017).

Another priority is that formulation be linked to exposure, and exposure to the pharmacodynamic effect. Earlier inclusion of pharmacokinetic measurements in animal and human studies is warranted wherever possible, as insufficient exposure could be a factor contributing to the lack of efficacy seen in clinical studies despite strong animal activity (Zhu et al., 2013; Li et al., 2018). Human micellar data confirm that formulation can markedly change systemic exposure (Chang et al., 2025), while experimental liver-fibrosis studies show that catalase-modified liposomes, HA-DOCA zein nanocolloids, dual-targeting PPC liposomes, and redox nanoparticles can alter delivery and selected efficacy measures (Wu et al., 2025; Wen et al., 2025; Li Y. et al., 2025; Nguyen et al., 2026). Clinical trials should also move toward fibrosis-centered endpoints, including histological stage, elastography, validated serum markers, collagen-turnover measures, and tissue-level mechanistic readouts when ethically and technically feasible (Kheong et al., 2017; Navarro et al., 2019). The prognostic importance of fibrosis in MASLD further supports this emphasis (Lam et al., 2024; Younossi et al., 2026). A practical benchmark is provided by human pharmacokinetics: conventional oral dosing generally produces submicromolar free silybin exposure (Zhu et al., 2013). Mechanistic studies should therefore report the concentrations tested alongside available information on unbound plasma or tissue exposure. For emerging mechanisms such as succinate signaling, FTH1/ferroptosis, and ANXA6, the lack of directly comparable human target-tissue concentrations currently prevents firm exposure-response bridging (Yang et al., 2025; Hu et al., 2025; Guan et al., 2026).

New target-level mechanisms should be treated as hypotheses requiring confirmation, not as established explanations. In particular, the FTH1-ferroptosis mechanism described in renal fibrosis and the ANXA6-PI3K/AKT/mTOR mechanism reported in pulmonary fibrosis should be independently replicated, related to achievable tissue concentrations, and evaluated in human-relevant systems (Hu et al., 2025; Guan et al., 2026). Clinically, silymarin may ultimately be more plausible as an adjunctive fibro-inflammatory modulator than as monotherapy for advanced fibrosis. Combinations with metabolic treatment, anti-inflammatory strategies, or locally delivered therapy may therefore deserve greater attention than stand-alone systemic treatment.

The proposed translational framework for future development of silymarin-based antifibrotic strategies is shown in Figure 2.

**FIGURE 2:**
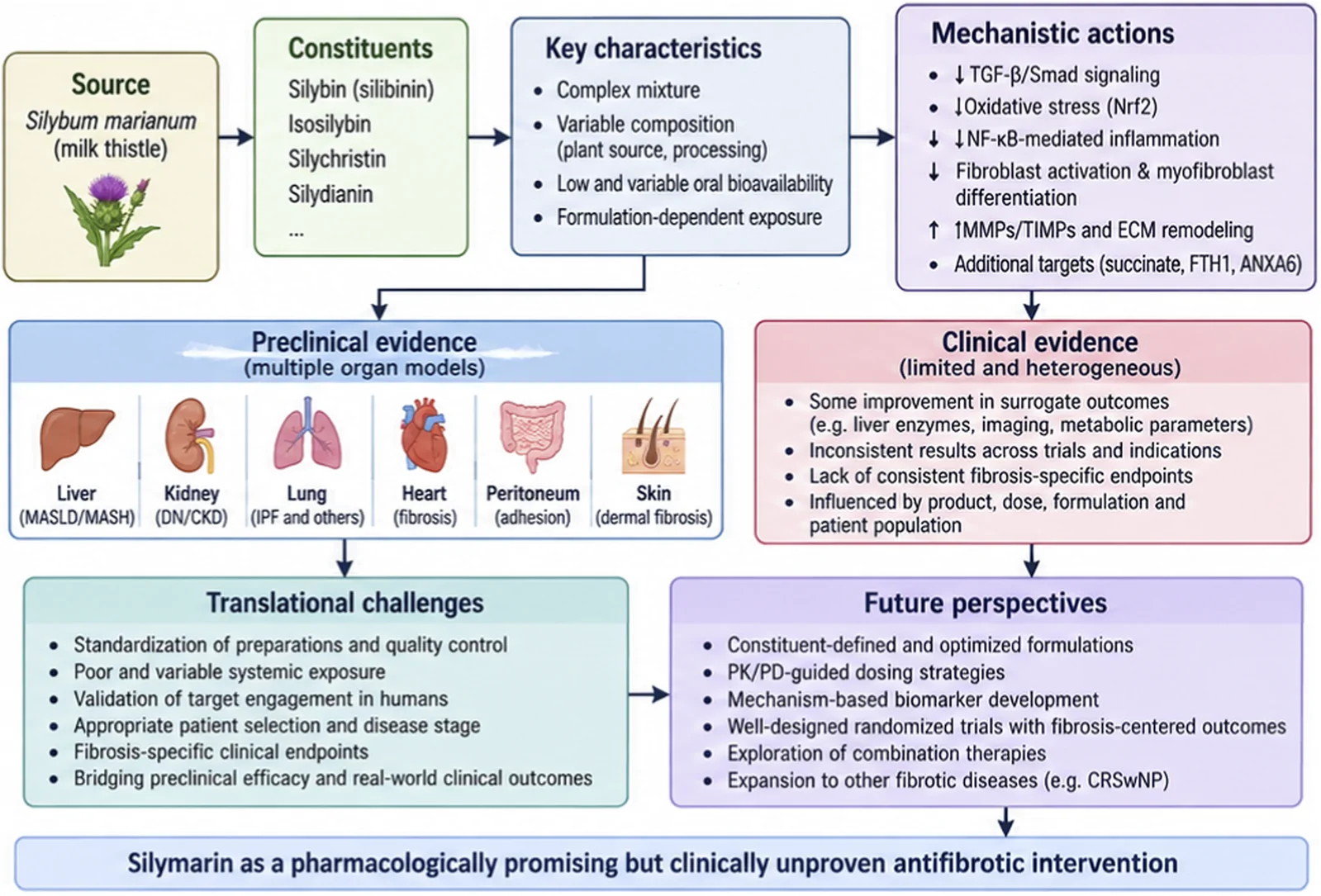
Translational framework for development of silymarin-based fibrosis strategies. The framework separates preclinical antifibrogenic activity from clinically demonstrated fibrosis modification. Priorities include explicit definition of the tested constituent or formulation, chemical standardization, exposure-informed dosing, causal target validation, fibrosis-stage classification, and fibrosis-specific clinical outcomes. At present, no silymarin component or formulation has demonstrated reproducible reversal of fibrosis in humans. Abbreviations: ALT, alanine aminotransferase; MASLD, metabolic dysfunction-associated steatotic liver disease; MDA, malondialdehyde; NAS, NAFLD activity score; NASH, nonalcoholic steatohepatitis; RCT, randomized controlled trial; TNF-α, tumor necrosis factor-α.
